# Monthly Record of Deaths in the City of Buffalo

**Published:** 1859-02

**Authors:** H. D. Garvin

**Affiliations:** Health Physician


ART. IX.—*Report of Mortality in Buffalo for the Month of Dec.,* 1858.
By H. D. Garvin, M. D., Health Physician.

| DISEASES. | No. | Males. | Females. | No Sex given. |
|---|---|---|---|---|
| Accident, | 4 | 2 | 2 | |
| Angina Maligna, | 1 | 1 | | |
| Apoplexy, | 1 | | | 1 |
| Asthma, | 2 | 2 | | |
| Bronchitis, | 1 | 1 | | |
| Cancer, | 2 | 1 | 1 | |
| Child Birth, | 2 | | 2 | |
| Consumption, | 18 | 6 | 12 | |
| Convulsions, | 6 | 4 | 2 | |
| Croup, | 3 | 1 | 2 | |
| Delirium Tremens, | 4 | 3 | 1 | |
| Debility, | 5 | 2 | 3 | |
| Diarrhœa, | 2 | 1 | 1 | |
| Disease of Brain, | 2 | 2 | | |
| " " Lungs, | 2 | 1 | 1 | |
| Dropsy, | 2 | 1 | 1 | |
| Enteritis, | 1 | 1 | | |
| Fever, Typhoid, | 3 | 3 | | |
| " Typhus, | 2 | 1 | | 1 |
| " Scarlet, | 1 | | | 1 |
| Gangrene, | 1 | 1 | | |
| Gastritis, | 2 | 1 | 1 | |
| Hooping Cough, | 1 | | 1 | |
| Inflammation of Lungs, | 16 | 8 | 8 | |
| " " Liver, | 1 | | 1 | |
| Intemperance, | 6 | 4 | 2 | |
| Marasmus, | 6 | 2 | 4 | |
| Meningitis, | 1 | 1 | | |
| Metritis, | 1 | | 1 | |
| Old Age, | 1 | 1 | | |
| Pyemia, | 1 | 1 | | |
| Rheumatism, | 1 | 1 | | |
| Small Pox, | 4 | 1 | 3 | |
| Still Born, | 5 | 3 | 2 | |
| Syphilis, | 2 | 1 | 1 | |
| Tabes Mesenterica, | 1 | 1 | | |
| Thrush, | 2 | 1 | 1 | |
| Ulceration of Bladder, | 1 | 1 | | |
| Unknown, | 8 | | | |
| Total, | 125 | 61 | 53 | 11 |

SEXES.

| | |
|---|---|
| Males, | 61 |
| Females, | 53 |
| Sex not given, | 11 |
| Total, | 125 |

### AGES.

| | | | |
|---|---|---|---|
| Still-born, | 5 | Between 20 years and 30 years, | 13 |
| 1 day, | 0 | " 30 " " 40 " | 20 |
| 1 day and 30 days, | 8 | " 40 " " 50 " | 5 |
| Between 1 month and 6 months, | 13 | " 50 " " 60 " | 11 |
| " 6 months and 12 " | 8 | " 60 " " 70 " | 4 |
| " 1 year " 3 years, | 14 | " 70 " " 80 " | 5 |
| " 3 " " 5 " | 3 | " 80 " " 90 " | 1 |
| " 5 " " 10 " | 4 | " 90 " " 100 " | 0 |
| " 10 " " 20 " | 4 | " 100 " | 0 |
| | 59 | | 59 |
| Ages not given, | 7 | | 59 |
| Total, | 125 | | |

### NATIVITIES.

| | | | |
|---|---|---|---|
| American, | 79 | French, | 3 |
| German, | 13 | Holland, | 0 |
| Irish, | 20 | Wales, | 0 |
| Canadian, | 0 | Prussian, | 0 |
| English, | 7 | Italian, | 1 |
| Scotch, | 0 | Country not given, | 2 |
| Total, | | | 125 |

*New Operation for Hare-Lip.*—Allen Duke makes this operation by paring the edges of the fissure obliquely from before backwards, and introducing the sutures, armed by two needles, just behind the skin, through the rest of the thickness of the lip. The removal of the upper suture is facilitated by bringing out the ends of the angle of the mouth and securing, with adhesive plaster.

When there is fissure of the jaw, the projecting portion is not completely, severed, but, just enough to admit of its being used to fill up the space, the edges being pared and sutures introduced before the lip is operated upon.

He reports, in the *Lancet*, four operations upon children, whose ages varied from six weeks to four months.—*The Peninsular and Independent Medical Journal.*

*Suffocation following the Operation for Hare-Lip.*—Dr. Jacobi, in a Report on Infantile Pathology and Therapeutics, in the *Montreal Chronicle*, says that Professor Busch has directed attention to the fact that the infants accustomed to breathe through a large abnormal opening, with the mouth closed, are sometimes liable to suffocation after the operation for Hare-Lip, by continuing to keep the mouth in that condition. Professor Volkman reports a fatal case of this kind in the *Monatschrift für Geburtskunde and Frauenkrankheiten.* The little patient was one year old. Dr. Gurlt has also seen similar cases.—*Ibid.*

